# Computerized margin and texture analyses for differentiating bacterial pneumonia and invasive mucinous adenocarcinoma presenting as consolidation

**DOI:** 10.1371/journal.pone.0177379

**Published:** 2017-05-18

**Authors:** Hyun Jung Koo, Mi Young Kim, Ja Hwan Koo, Yu Sub Sung, Jiwon Jung, Sung-Han Kim, Chang-Min Choi, Hwa Jung Kim

**Affiliations:** 1 Department of Radiology and Research Institute of Radiology, Asan Medical Center, University of Ulsan College of Medicine, Seoul, Republic of Korea; 2 Department of Infectious Diseases, University of Ulsan, Ulsan, Republic of Korea; 3 Department of Infectious Diseases, Asan Medical Center, University of Ulsan College of Medicine, Seoul, Republic of Korea; 4 Pulmonary and Critical Care Medicine, and Department of Oncology, Asan Medical Center, University of Ulsan College of Medicine, Seoul, Republic of Korea; 5 Department of Clinical Epidemiology & Biostatistics, Asan Medical Center, University of Ulsan College of Medicine, Seoul, Republic of Korea; Toranomon Hospital, JAPAN

## Abstract

Radiologists have used margin characteristics based on routine visual analysis; however, the attenuation changes at the margin of the lesion on CT images have not been quantitatively assessed. We established a CT-based margin analysis method by comparing a target lesion with normal lung attenuation, drawing a slope to represent the attenuation changes. This approach was applied to patients with invasive mucinous adenocarcinoma (n = 40) or bacterial pneumonia (n = 30). Correlations among multiple regions of interest (ROIs) were obtained using intraclass correlation coefficient (ICC) values. CT visual assessment, margin and texture parameters were compared for differentiating the two disease entities. The attenuation and margin parameters in multiple ROIs showed excellent ICC values. Attenuation slopes obtained at the margins revealed a difference between invasive mucinous adenocarcinoma and pneumonia (*P*<0.001), and mucinous adenocarcinoma produced a sharply declining attenuation slope. On multivariable logistic regression analysis, pneumonia had an ill-defined margin (odds ratio (OR), 4.84; 95% confidence interval (CI), 1.26–18.52; *P* = 0.02), ground-glass opacity (OR, 8.55; 95% CI, 2.09–34.95; *P* = 0.003), and gradually declining attenuation at the margin (OR, 12.63; 95% CI, 2.77–57.51, *P* = 0.001). CT-based margin analysis method has a potential to act as an imaging parameter for differentiating invasive mucinous adenocarcinoma and bacterial pneumonia.

## Introduction

The International Association for the Study of Lung Cancer, the American Thoracic Society, and the European Respiratory Society have recommended changing the term “mucinous bronchioloalveolar carcinoma” to “mucinous adenocarcinoma”[1]. This entity differs from the mucinous type of adenocarcinoma in situ or minimally invasive adenocarcinoma by having a focus of invasion >5-mm and size >3-cm [1]. Because of the bronchogenic dissemination and air space spread of the invasive mucinous adenocarcinoma, it is not easily differentiated radiologically from pneumonia [2–4]. Even in terms of clinical characteristics, the substantially overlapping conditions are always challenging for clinicians and radiologists. If we could differentiate malignancy and pneumonia in a quantitative manner, it would benefit decisions on appropriate treatment.

Previous quantitative CT studies have analyzed the internal texture of nodules and mass [5–7]. Radiologists have typically used margin characteristics based on routine visual analysis; however, the attenuation changes at the margin of the lesion in CT images have not been quantitatively assessed. In this study, we investigated visual and texture analyses to compare invasive mucinous adenocarcinoma presenting as consolidation and bacterial pneumonia. Also, we established a margin analysis method by comparing target lesions with normal lung attenuation, drawing a linear slope to represent the attenuation changes.

## Materials and methods

This retrospective study was approved by Asan Medical Center institutional review board (approval 2015–1312) with waiver of the requirement for patients’ informed consent.

### Study population and design

**Fig 1:**
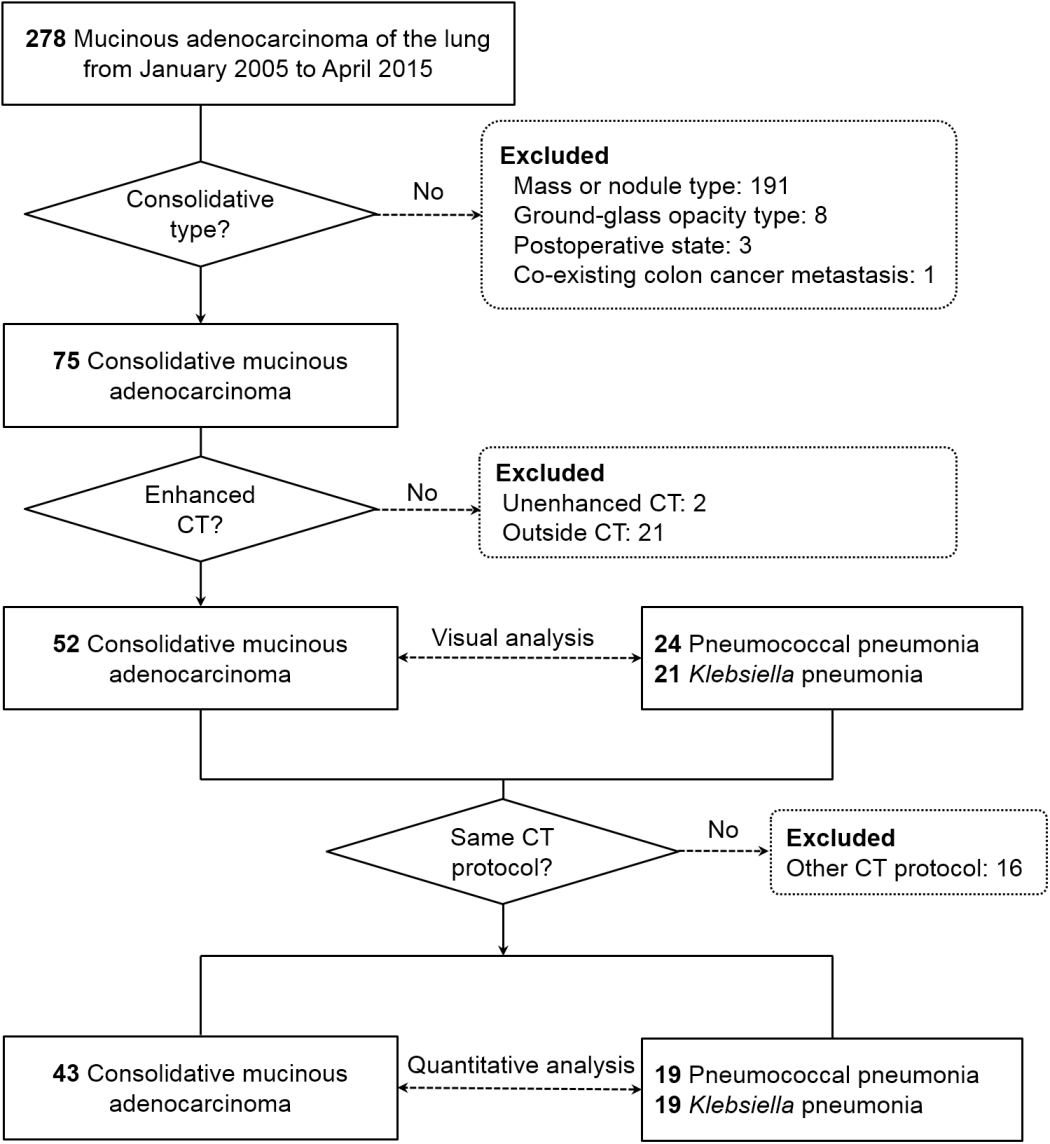
Flowchart of patients.

We searched the pathology database at our institution from Jan 2005 to April 2015 and found 278 consecutive patients who were pathologically diagnosed as having invasive mucinous adenocarcinoma of the lung. Patients who have other possible causes of consolidation including infection or other tumors were excluded. The diagnosis was made by bronchoscopic biopsy or bronchoalveolar lavage (n = 11), wedge resection or lobectomy (n = 103), and fluoroscopic or CT-guided transthoracic core needle biopsy (n = 164). To identify the invasive mucinous adenocarcinoma showing consolidation on CT, 203 patients were excluded for the following reasons: mass or nodule type (n = 191), ground-glass opacity (GGO) type (n = 8), unavailable preoperative CT scan (n = 3), and co-existing lung metastasis from colon cancer (n = 1). Unenhanced CT (n = 2) and outside hospital CT examinations (n = 21) were also excluded so that finally 52 patients with invasive mucinous adenocarcinoma remained in the study (Fig 1).

In addition, we searched culture databases and electronic medical records to identify consecutive patients with proven pneumococcal or *Klebsiella* pneumonia who had enhanced CT examinations between Jan 2005 and Dec 2014. The diagnosis of pneumonia was based on bacteremia (n = 42) or culture of expectorated or endotracheal aspiration sputum (n = 41). In our hospital, we routinely obtain three blood culture bottles from a patient who is suspected to have pneumonia. If there were more than two bottles of positive blood cultures, it was considered as true positive result. Also, we routinely perform repeated blood culture or sputum study if it is clinically suspected as contamination. Sputum cultures in 41 patients were performed with adequate specimens (> 25 polymorphonuclear cells per low power field) and < 10 squamous epithelial cells per low power field). Finally, 45 pneumonia patients showing air-space consolidation on enhanced CT were included in the study. Clinical data including patient demographics, symptoms, laboratory findings, and date of initial CT scan were collected using our electronic medical record system. The initial CT scans were analyzed visually and quantitatively. To obtain the parameters of CT-based texture and margin analyses, 1-mm slice thickness CT images were used, and 16 patients without 1-mm thickness CT images were excluded. In 81 CT examinations obtained with identical CT settings, the margins of 11 lesions could not be drawn because they abutted a fissure or adjacent structures or because of atelectasis (n = 9) or underlying severe emphysema (n = 2). Forty cases of invasive mucinous adenocarcinoma and 30 with pneumonia were used to compare the margin-based analysis (Fig 1).

### CT image acquisition

Chest CT examinations were made using two CT scanners: SOMATOM Sensation 16 (n = 21; Siemens Healthcare, Erlangen, Germany) and SOMATOM Definition AS (n = 64; Siemens Healthcare). The scan parameters for the CT scanners were identical to the standard setting for thorax: 120 kV, 100 effective mA with dose modulation, 3 / 5 mm thickness reconstruction interval without a gap by standard algorithm, and 1 mm thickness with 5 mm gaps by high-frequency algorithm. In all cases, 100 mL of intravenous iopromide 300 (300 mg I/mL; Ultravist, Bayer Pharma, Berlin, Germany) was administered at a rate of 2.5 mL/s using a power injector to obtain contrast enhancement images; an enhanced CT scan was obtained after a 50 s delay. All CT scans were obtained on axial images and reconstructed as coronal images. Mediastinal (width, 450 Hounsfield unit (HU); level, 50 HU), lung (width, 1500 HU; level, -700 HU) and bone (width, 1000 HU; level 200 HU) windows were reviewed on the picture archiving and communication system (PACS).

### CT visual analysis

Two independent thoracic radiologists (M.Y.K., 20 years’ experience in thoracic radiology; H.X., fellowship-trained thoracic radiologist), blinded to the clinical and image analysis data except for the fact that the patient was included in the study, evaluated CT images using a PACS workstation. The final results of the visual analyses were reached in consensus. CT characteristics were thoroughly reviewed: 5-lobar location, lesion homogeneity referent to muscle, ill-defined margin, presence of cavity formation, internal bubble lucency, necrosis, open bronchus or vessel sign resulting from pulmonary vessels preserved within the consolidative lesion, fissure bulging, local pleural thickening, pleural retraction, adjacent GGO foci, concurrent bronchial wall thickening, interlobular septal thickening, centrilobular nodules (<1 cm), macronodules (>1 cm diameter), multiplicity, and presence of effusion [8; 9]. The conclusions of formal reading of the CT scans were recorded and divided into three categories: correct as first diagnosed, suggested from differential diagnosis, and incorrect diagnosis. CT visual analysis findings of all patients are included in S1 File. The minimal data for CT margin and texture analyses obtained from one ROI per patient are included in S2 File.

### CT margin analysis

**Fig 2:**
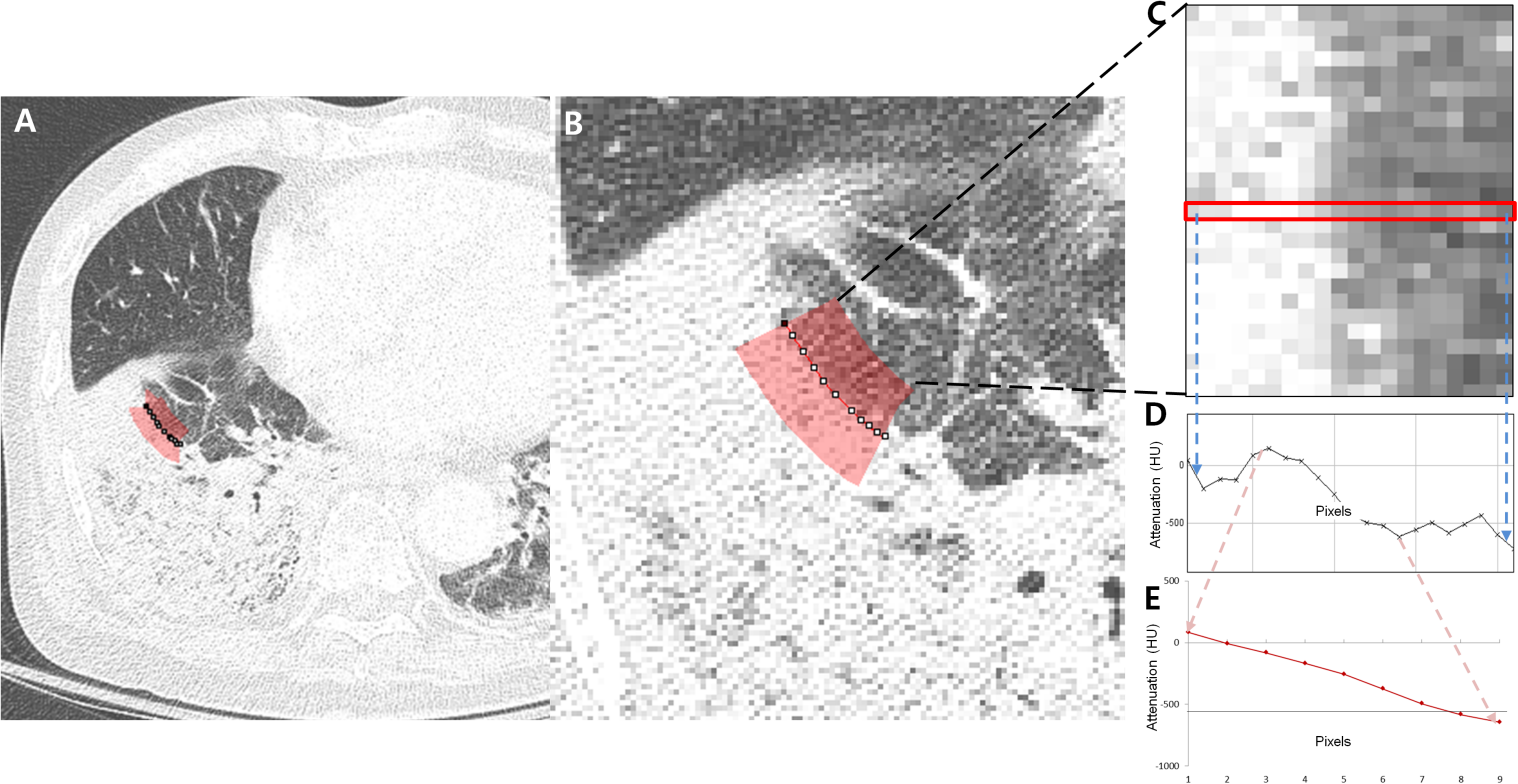
An example of margin-based analysis for obtaining the attenuation slope from lesion to normal lung in pneumococcal pneumonia. (A, B) Axial CT image shows an area of margin (red) selected for obtaining the attenuation transition from lesion to normal lung. (C) Pixels in the selected area in (B) were plotted as rectangular boxes with multiple pixels showing the attenuation transition from consolidation (left side) to normal lung (right side). (D) The graph shows the attenuation changes from consolidation (left side) to normal lung (right side) among one of the pixel lines (red box from C). (E) Average attenuation changes within the nine pixels from the center of the highest slope in the selected area plotted from the consolidation (left side) to normal lung (right side). HU, Hounsfield unit.

To obtain CT-based margin features, the digital imaging and communications in medicine (DICOM) data from CT scans were computed using an in-house developed software based on plug-in package for ImageJ (Bethesda, Maryland; http://rsbweb.nih.gov/ij/). To reduce selection bias, two ROIs (>20 pixels) with direction perpendicular to the margin of lesion were drawn at random (Fig 2). The selected areas were plotted as linear pixels and the attenuation slope was obtained from each straight line. To show the transition at the margin, the ROI should include both normal lung parenchyma and a part of the target lesion; thus, not all boundary regions could be selected. For example, if a target lesion was located near the fissure or chest wall, the part of the boundary near the adjacent structures could not reveal the transition to normal lung. Similarly, parts of a lesion with adjacent vessels or bronchi could not be used for the same reason.

**Fig 3:**
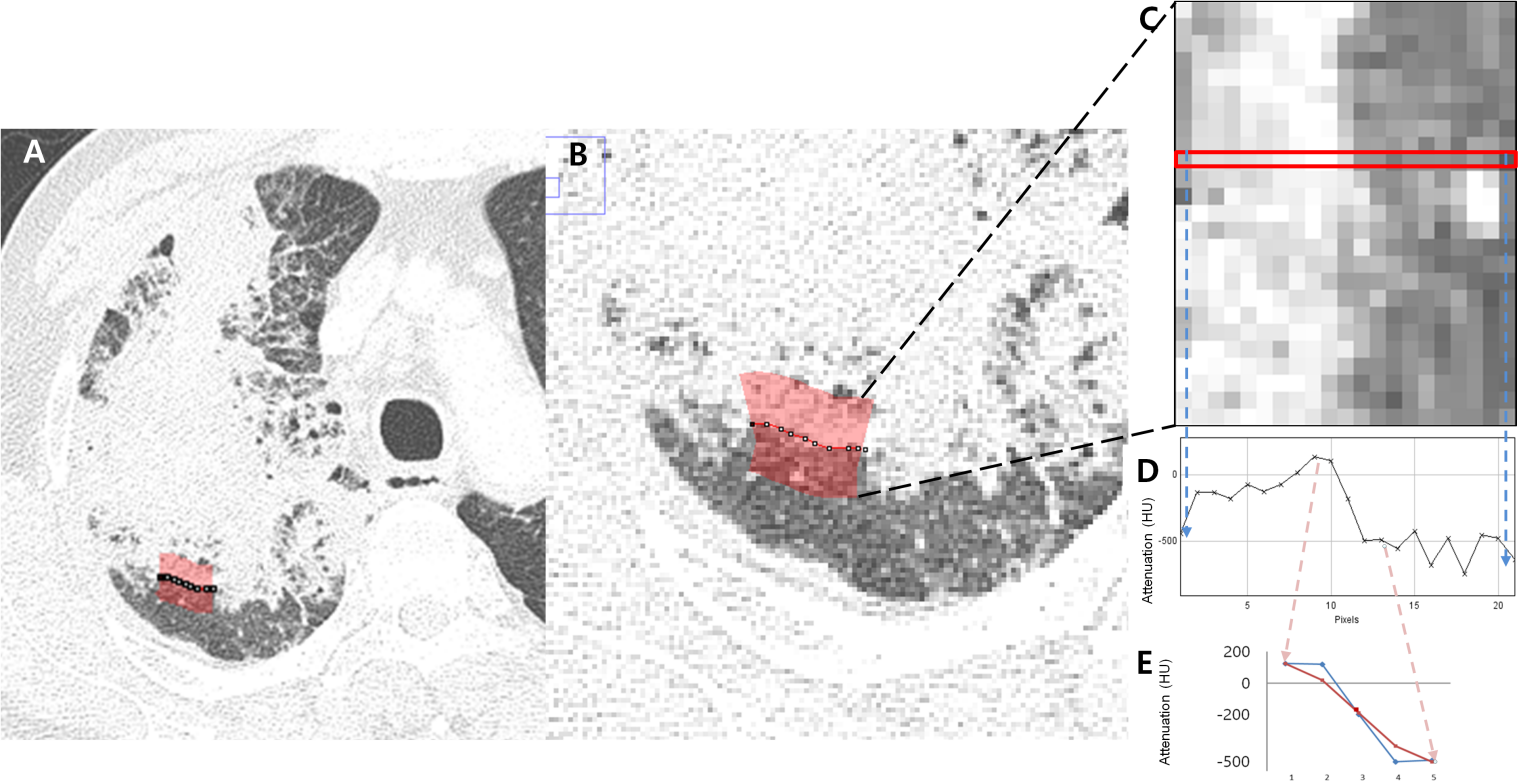
An example of margin-based analysis for obtaining the attenuation slope from lesion to normal lung in invasive mucinous adenocarcinoma. (A, B) Axial CT image shows an area of margin (red) selected for obtaining the attenuation transition from lesion to normal lung. (C) Pixels in the selected area in (B) were plotted as rectangular boxes with multiple pixels showing the attenuation transition from consolidation (left side) to normal lung (right side). (D) The graph shows the attenuation changes from consolidation (left side) to normal lung (right side) among one of the pixel lines (red box from C). (E) Average attenuation changes within the five pixels from the center of the highest slope in the selected area plotted from the consolidation (left side) to normal lung (right side). HU, Hounsfield unit.

In the ROI, pixel attenuation from the lesion to normal lung was plotted as a graph, and the mean and maximal slopes among the pixels were obtained (Figs 2D and 3D). The selected pixel numbers were 5, 7 and 9 pixels, and average values of multiple slopes of the pixel attenuations were also calculated (Figs 2E and 3E). The less steeply the attenuation slope declines from lesion to normal lung, the more the margin is regarded as fading out gradually. In contrast, the steeper the slope, the more distinct and rapid the attenuation of the transition from lesion to normal lung.

The correlations of the margin-based features obtained from two ROIs were obtained, and the mean values of the two ROIs were used as final values for a target lesion. The ROIs for margin-based analysis were drawn by two radiologists (H.J.K. and H.X. with 2 years of experience in thoracic radiology) in consensus and confirmed by an experienced radiologist (M.Y.K.). ROIs were semi-automatically selected by clicking a part of a lesion. Margin-based features were automatically computed to obtain the attenuation slopes from lesion to normal lung. The slope represents the gradient of attenuation transition at the margin of a target lesion. The two ROIs were drawn in 1-mm CT images, to avoid summation of attenuation values when using thicker slices.

### CT texture analysis

**Fig 4:**
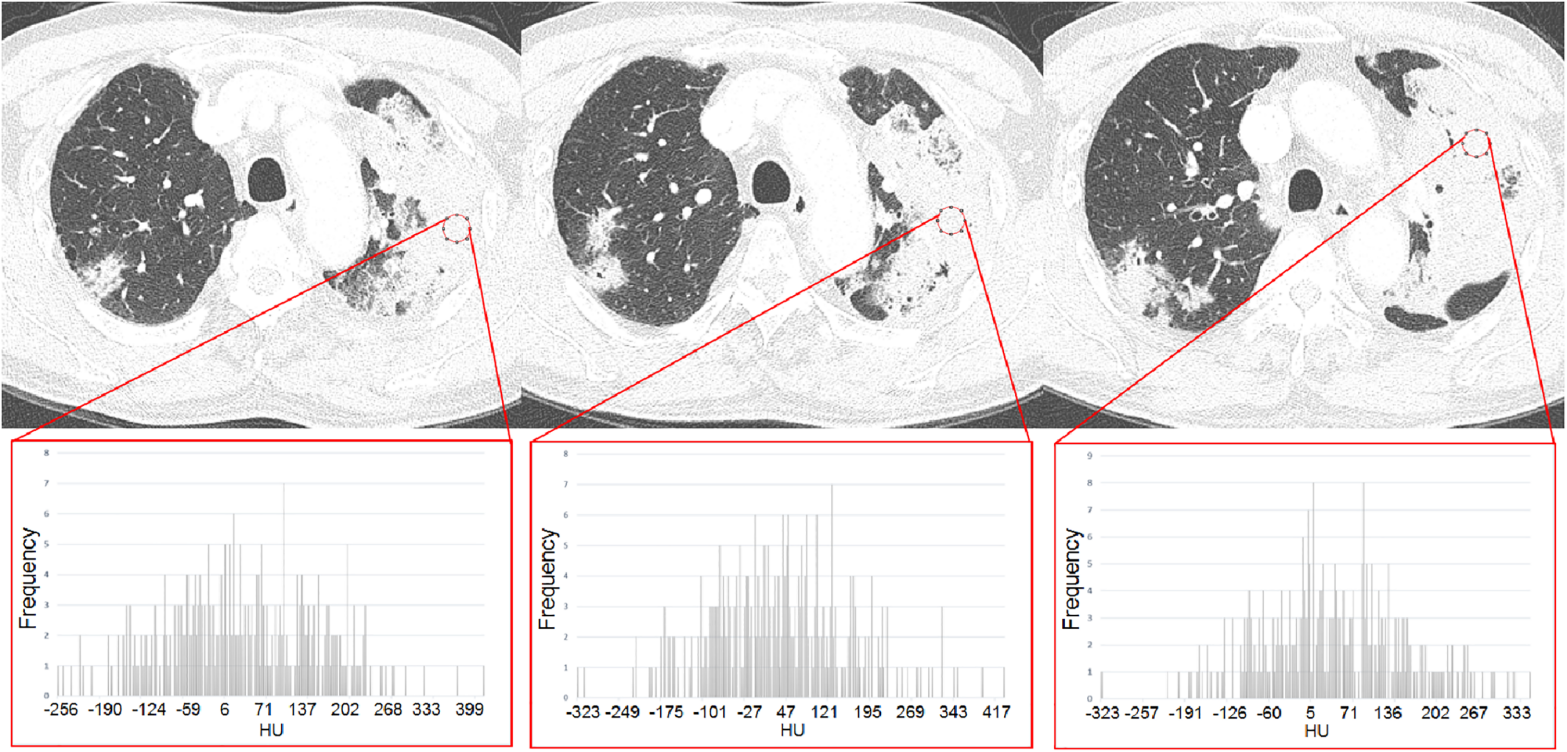
Region of interests (ROIs) for CT texture analysis in a patient with invasive mucinous adenocarcinoma. CT images of 1-mm slice thickness with three round ROIs in a consolidative lesion. Histograms show the distributions of CT attenuation (HU, Hounsfield unit) of the ROIs on the horizontal axis. The vertical axis of the histograms shows the frequencies of attenuation of the lesion.

To obtain CT texture-based features, DICOM data of CT scans were loaded and computed using ImageJ program. To reduce selection bias, three round ROIs (20 pixels) were randomly drawn within representative slices of consolidations (Fig 4). The correlation of texture features obtained from the ROIs was calculated and the mean value of the ROIs was used as a final value for the target lesion. The ROIs for texture analysis were drawn by two radiologists (H.J.K. and H.X.) in consensus and confirmed by an experienced radiologist (M.Y.K.). ROIs were semi-automatically selected by clicking parts of a lesion on 1-mm thickness CT images. In selection of ROIs, pulmonary vessels, bronchus, cavities, and adjacent chest wall were avoided. Texture parameters measured were attenuation, skewness, kurtosis, and entropy. Skewness was defined as the degree of asymmetry of a pixel distribution and calculated as $E\;\left[{\left(\frac{X-\mu }{\sigma }\right)}^{3}\right]$, where X = attenuation, *σ* = mean of attenuation, and *μ* = standard deviation of attenuation. Kurtosis was defined as the magnitude of the pixel distribution and calculated as $k=\frac{{E(x-\mu )}^{4}}{\sigma^{4}}-3$, where x = attenuation, *σ* = mean of attenuation, and *μ* = standard deviation of attenuation. Entropy was described as degree of randomness in the ROI, and noted as $\sum_{i=1}^{n}p(x_{i})\;\log\;p(x_{i})$, where, *x*_*i*_ = frequency from histogram of ROI and *p*(*x*_*i*_) = probability on histogram. The values were derived automatically using ImageJ software.

### Statistical analysis

Categorical variables are given as numbers and percentages, continuous variables as mean and standard deviation. Student *t*-test and the Chi square test were used to compare patient characteristics, CT visual analysis, texture and margin analyses results between invasive mucinous adenocarcinoma and bacterial pneumonia. Inter-value correlations among ROIs were obtained from intraclass correlation coefficients (ICCs). Receiver operating curves to demonstrate the diagnostic performances of the attenuation slopes at the margin of lesions for differentiating invasive mucinous adenocarcinoma and pneumonia were obtained. To classify the patients into two groups using a binary slope cut-off, one of the receiver operating curves with the highest area under the curve was selected to define the cut-off value of the slope with optimal sensitivity and specificity. Univariate and multivariate logistic regression analyses were used to identify independent factors among the results for CT visual assessment, CT texture, and binary slope for differentiating invasive mucinous adenocarcinoma and pneumonia. SPSS version 21.0 (SPSS Inc., Chicago, IL, USA) was used for all statistical analyses.

## Results

### Clinical characteristics

Patients’ clinical characteristics, including smoking history, concomitant chronic respiratory disease, and co-morbidities such as chronic heart failure, diabetes, and liver cirrhosis, together with the initial presenting symptoms and laboratory findings are shown in Table 1. The duration from the initial onset of symptoms in patients with invasive mucinous adenocarcinoma was relatively long compared with the pneumonia patients (*P* <0.001). Symptoms such as fever, chest pain, sputum, and dyspnea were more frequently observed in pneumonia patients. White blood cell counts and C-reactive protein levels were also significantly higher in pneumonia patients. However, symptom durations, white blood cell counts, and C-reactive protein levels overlapped in parts of the population of the two groups.

**Table 1:** Clinical characteristics of patients with pathologically confirmed consolidative mucinous adenocarcinoma and bacterial pneumonia.

	Mucinous adenocarcinoma	Bacterial pneumonia	*P* value
No. of patients	52	45	
Age	67.3 ± 10.8 [46–91]	62.6 ±17.2 [15–88]	0.11
Male	27 (51.9)	32 (71.1)	0.05
Smoking			0.34
Never smoked	27 (51.9)	19 (42.2)	
Smoker	25 (48.1)	26 (57.8)	
Pack year (smoker)	30.4 ± 22.5 [2–106]	33.1 ± 27.4 [1–120]	0.70
Co-morbidity			
Chronic respiratory disease	3 (5.7)	5 (11.1)	0.47
Congestive heart failure	0 (0)	2 (4.4)	0.21
Diabetes	14 (26.9)	15 (33.3)	0.49
Liver cirrhosis	1 (1.9)	2 (4.4)	0.60
Other malignancy	4 (7.7)	16 (35.5)	<0.001
Symptom			
Duration (days) ^‡^	116.8 ± 179.8 [3–1080]	4.1 ± 4.9 [0–30]	<0.001
Asymptomatic	8 (15.3)	1 (2.2)	
Fever	2 (3.8)	29 (64.4)	<0.001
Cough	35 (67.3)	37 (82.2)	0.09
Chest pain	3 (5.8)	12 (26.7)	0.01
Sputum	31 (59.6)	38 (84.4)	0.01
Dyspnea	13 (25.0)	28 (62.2)	<0.001
Drowsy mentality	0 (0)	3 (6.7)	0.10
Laboratory findings			
White blood cells x10^3^	6.9 ± 2.3 [3.4–12.8]	12.4 ± 10.2 [0.1–46.1]	0.001
C-reactive protein	3.3 ± 6.5 [0.1–30.2]	22.9 ± 12.8 [0.1–53.4]	<0.001
Blood urea nitrogen	15.2 ± 5.7 [7–35]	25.8 ± 14.3 [5–68]	<0.001
Creatinine	0.8 ± 0.2 [0.5–1.5]	1.1 ± 0.5 [0.4–2.67] ^†^	<0.001
CURB-65 (0–5)	1.9 ± 1.3	0.9 ± 1.0	< 0.001

Note. Unless otherwise indicated, data are number of patients (%). Data are mean ± standard deviation [range].

^‡^Duration from symptom onset.

^†^Five patients with serum creatinine levels >1.7 mg/dL were hydrated before and after contrast enhanced CT examination and monitored for creatinine levels after CT examination. CURB-65, pneumonia severity index.

### CT visual analysis

The results of visual assessment of CT findings in the study patients were noted in Table 2. Of the 52 CT scans showing invasive mucinous adenocarcinoma, 44% were correctly diagnosed as lung cancer at first impression; lung cancer and pneumonia were suggested as differential diagnoses in a further 42%; the remaining seven cases (14%) were misdiagnosed as pneumonia. Among the 45 cases of pneumonia, 35 (78%) were correctly diagnosed as pneumonia at first impression; nine cases were suggested as pneumonia or lung cancer as differential diagnoses; one case was misdiagnosed as fungal infection.

**Table 2:** CT visual assessment of invasive mucinous adenocarcinoma and bacterial pneumonia.

		Invasive Mucinous adenocarcinoma	Bacterial pneumonia	
No. of patients		52	45	
CT reading	Correct	23 (44.2)	35 (77.8)	
	Differential	22 (42.3)	9 (20.0)	
	Missed	7 (13.5)	1 (2.2)	
Location*	Right upper lobe	3 (5.8)	14 (31.1)	
	Right middle lobe	1 (1.9)	7 (15.6)	
	Right lower lobe	26 (50)	9 (20.0)	
	Left upper lobe	4 (7.7)	3 (6.7)	
	Left lower lobe	18 (34.6)	12 (26.7)	
CT visual analysis		Invasive Mucinous adenocarcinoma	Bacterial pneumonia	*P* value
Heterogenous attenuation		11 (21.2)	16 (35.6)	0.11
Ill-defined margin		13 (25.0)	33 (73.3)	<0.001
Cavity		17 (32.7)	10 (22.2)	0.25
Internal bubble lucency		31 (59.6)	13 (28.9)	0.002
Necrosis		9 (17.3)	12 (26.7)	0.26
Open bronchus sign		42 (80.8)	39 (86.7)	0.44
Open vessel sign		32 (61.5)	45 (100)	<0.001
Fissure bulging		14 (26.9)	28 (62.2)	< 0.001
Local pleural thickening		6 (11.5)	2 (4.4)	0.28
Pleural retraction		12 (23.1)	2 (4.4)	0.01
Ground-glass opacity foci		12 (23.1)	32 (71.1)	<0.001
Bronchial wall thickening		2 (3.8)	12 (26.7)	0.003
Interlobular septal thickening		1 (1.9)	2 (4.4)	0.60
Centrilobular nodules		10 (19.2)	15 (33.3)	0.11
Macronodules		24 (46.2)	2 (4.4)	< 0.001
Multiple consolidation		15 (28.8)	29 (64.4)	< 0.001
Pleural effusion		5 (9.6)	19 (42.2)	< 0.001

Note. Unless otherwise indicated, data are numbers of patients (%). *If lesions were multiple, the location of the largest lesion was recorded.

When compared with invasive mucinous adenocarcinoma, pneumonia had ill-defined margins (73%) and surrounding GGO (71%) (*P*<0.001). Open vessel signs and bulging fissures were noted more frequently in pneumonia (*P*<0.001). Bronchial wall thickening (27%) and pleural effusion (42%) were also common in pneumonia (*P*<0.05). Internal bubble lucency (60%) and pleural retraction (23%) were more commonly noted in invasive mucinous adenocarcinoma (*P*<0.05). Multiple consolidations were noted more often in pneumonia (64%) (*P*<0.001).

### CT margin analysis

**Fig 5:**
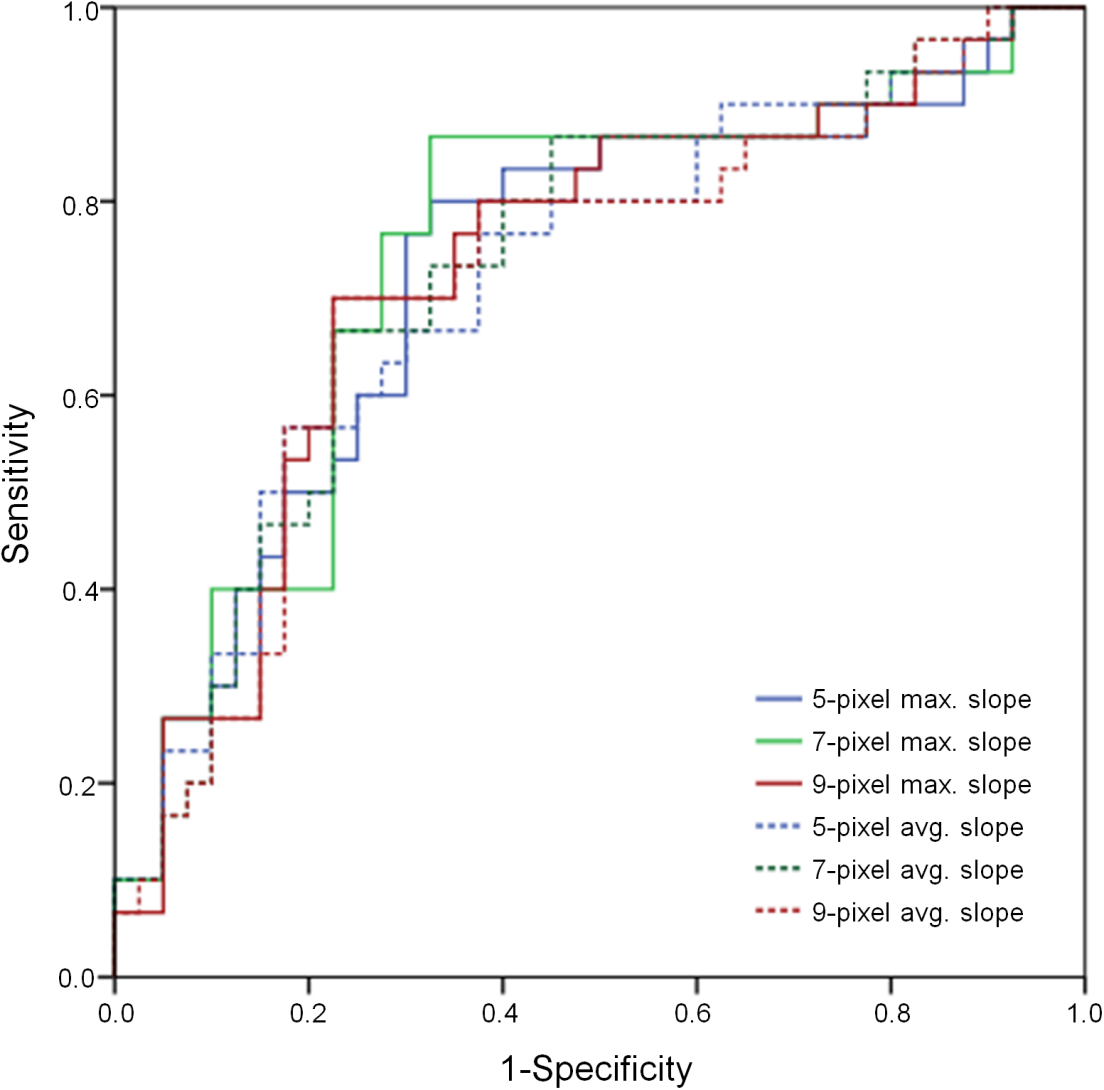
Receiver operating curves showing the diagnostic performance of the attenuation slope at the margin of lesions for differentiating between invasive mucinous adenocarcinoma and bacterial pneumonia manifesting as an air space consolidation. The C-indices for the graphs are 0.72–0.75, and the highest value is noted at the 7-pixel maximal slope. At a cut-off slope value of -154.21, sensitivity was 86.7% and specificity was 67.5% for the diagnosis of invasive mucinous adenocarcinoma using the 7-pixel maximal slope. max., maximal; avg., average.

The maximum attenuation transition slopes at the margin of the 5-pixel, 7-pixel, and 9-pixel distributions on histograms were automatically calculated and the average values of the slopes were obtained. Inter-ROI agreements for slopes were high 0.82–0.85 (*P*<0.001) (Table 3). In comparisons of invasive mucinous adenocarcinoma and pneumonia, all measured slopes of the variable pixel numbers at the margins were significantly different, and invasive mucinous adenocarcinoma had slopes that declined more steeply than those of pneumonia (Table 4). In the receiver operating curves of attenuation slopes to differentiate the two disease entities, the C-indices for the graphs were 0.72–0.75 and the highest value was noted for the 7-pixel maximal slope (Fig 5). At a cut-off slope value of -154.21, sensitivity of 86.7% and specificity of 67.5% were noted for the diagnosis of invasive mucinous adenocarcinoma using the 7-pixel maximal slope. Using that cut-off value of -154.21, all patients were classified into two groups. In multivariable logistic regression analysis for CT visual assessment and binary slope, an ill-defined margin (odds ratio (OR), 4.84; 95% confidence interval (CI), 1.26–18.52; *P* = 0.02), GGO (OR, 8.55; 95% CI, 2.09–34.95; *P* = 0.003), and binary slope (OR, 12.63; 95% CI, 2.77–57.51, *P* = 0.001) were independent factors differentiating invasive mucinous adenocarcinoma and bacterial pneumonia (Table 5).

**Table 3:** CT texture and margin analyses of invasive mucinous adenocarcinoma and bacterial pneumonia.

	ICC (*P* value)	Mucinous adenocarcinoma	Bacterial pneumonia	^*^P value
Mean attenuation (HU)	0.96 (<0.001)	36.0 ± 38.6	43.8 ± 57.3	0.47
Skewness	0.54 (<0.001)	-0.1 ± 0.4	- 0.004 ± 0.1	0.05
Kurtosis	-0.06 (0.610)	0.4 ± 1.5	- 0.005 ± 0.1	0.07
Entropy	0.79 (<0.001)	6.9 ± 0.1	6.9 ± 0.2	0.35
5-pixel max. slope	0.85 (<0.001)	-209.7 ± 39.0	-176.6 ± 41.2	<0.001
7-pixel max. slope	0.85 (<0.001)	-156.5 ± 25.4	-133.8 ± 27.3	<0.001
9-pixel max. slope	0.85 (<0.001)	-124.4 ± 18.3	-108.7 ± 19.7	<0.001
5-pixel avg. slope	0.82 (<0.001)	-191.4 ± 42.5	-156.4 ± 44.2	<0.001
7-pixel avg. slope	0.82 (<0.001)	-148.2 ± 28.0	-124.7 ± 29.2	<0.001
9-pixel avg. slope	0.84 (<0.001)	-119.4 ± 19.7	-102.8 ± 21.1	<0.001

Note.

*The differences of CT texture and margin parameters between invasive mucinous adenocarcinoma and bacterial pneumonia were calculated using Student t-test. HU, Hounsfiled unit; ICC, intraclass correlation coefficient among regions of interest per lesion; max., maximal; avg., average.

**Table 4:** Univariate analysis of CT margin-based analysis for differentiation of invasive mucinous adenocarcinoma and bacterial pneumonia.

1-mm CT images	*P* value	OR	95% CI
5-pixel max. slope	0.003	1.02	1.01–1.04
7-pixel max. slope	0.002	1.03	1.01–1.06
9-pixel max. slope	0.003	1.05	1.02–1.08
5-pixel avg. slope	0.003	1.02	1.01–1.03
7-pixel avg. slope	0.003	1.03	1.01–1.05
9-pixel avg. slope	0.003	1.04	1.01–1.07

Note. A decrease in slope by 1° increased the risk of bacterial pneumonia as noted odds ratio for slopes. CI, confidence interval; HU, Hounsfield unit; OR, odds ratio; max., maximal; avg., average.

**Table 5:** Univariate and multivariable analyses of CT visual assessment and binary slope for differentiation of invasive mucinous adenocarcinoma and bacterial pneumonia.

	Univariate			Multivariable		
	*P* value	OR	95% CI	*P* value	OR	95% CI
Ill-defined margin	< 0.001	6.89	2.62–18.14	0.02	4.84	1.26–18.52
Bubble lucency	0.006	0.28	0.11–0.70			
Open vessel sign	0.99					
Fissure bulging	0.005	3.78	1.50–9.51			
Ground-glass opacity	<0.001	6.75	2.58–17.69	0.003	8.55	2.09–34.95
Pleural retraction	0.04	0.19	0.04–0.95			
Binary slope*	<0.001	13.5	3.89–46.81	0.001	12.63	2.77–57.51

Note.

*Patients were classified into two groups according to the value of the 7-pixel maximal slope, using a cut-off slope value of -154.21. CI, confidence interval; OR, odds ratio.

### CT texture analysis

Using 81 CT examinations (43 invasive mucinous adenocarcinoma, 38 bacterial pneumonia) obtained with identical CT settings, three ROIs per lesion were chosen. Inter-ROI agreement for attenuation was high in 1-mm thickness CT images (0.96, *P*<0.001). For skewness (0.54, *P*<0.001) and kurtosis (-0.06, *P* = 0.06), the agreements among the three internal ROIs were not good. On univariate logistic regression analysis, no CT texture parameters were significant in differentiating invasive mucinous adenocarcinoma and bacterial pneumonia.

## Discussion

Because of substantially overlapping clinical characteristics, including symptoms such as cough and sputum, differentiation of invasive mucinous adenocarcinoma and pneumonia is not always easy [2–4]. When invasive mucinous adenocarcinoma presents as multifocal lesions, it can mimic pneumonia, and delayed diagnosis with progression could result after treatment with antibiotics. Complicated bacterial pneumonia with resistance to antibiotics could also mimic malignancy. Previous studies for evaluating mucinous adenocarcinoma have used CT visual assessment [9–13]. If malignancy and pneumonia could be differentiated in a quantitative manner, both types of cases would benefit from it.

On visual analysis of CT findings, pneumonia showed an ill-defined margin (odds ratio (OR), 4.84; *P* = 0.02) and ground-glass opacity (OR, 8.55; *P* = 0.003). In bacterial pneumonia, adjacent GGO foci around the consolidation may indicate multifocal pneumonia or inflammatory lesions. Although invasive mucinous adenocarcinoma also could present GGO by endogenous aspiration of mucin from the tumor cells, GGO was likely to be noted more frequently in pneumonia patients. Moreover, the attenuation slope at the margin of consolidation lesion in pneumonia had gradually declining attenuation (OR, 12.63; *P* = 0.001) compared to invasive mucinous adenocarcinoma.

Quantitative CT analysis has been developed in terms of shape-, texture-, and margin-based features. Shape-based parameters such as diameter, area, sphericity or lobulation of lesions are computed from 2-dimensional (D) or 3-D segmentation analysis [14–16]. Texture-based features such as skewness, kurtosis, and entropy are also used to differentiate malignancy or to monitor treatment responses [5; 17]. Several studies suggest margin-based features such as sharpness, acutance, and histogram spread of the average gradient (HSAG) of lesions to differentiate malignant and benign nodules [18; 19]. However, sharpness and acutance provide only boundary information without information on the continuous attenuation changes from lesion to normal lung, and HSAG shows only the average value of all boundary pixels of lesions.

Our study suggests that attenuation slopes are strong imaging markers for differentiation of invasive mucinous adenocarcinoma and bacterial pneumonia manifesting as air space consolidation. Because the margin is the battlefield of human body against cancer or pneumonia, margin sharpness and the attenuation distribution of the lesions are highly important and correlate well in discriminating tumor and inflammation on visual CT assessment. By measuring the attenuation slope at the margin of lesions, our findings introduce CT margin analysis as a potential biomarker for differentiating invasive mucinous adenocarcinoma and pneumonia manifesting as air space consolidation. Bacterial pneumonia has an ill-defined margin with large gray zone; in contrast, consolidative malignancy has a relatively distinct attenuation difference compared with normal lung, with a narrow gray zone at the margin. This may be because bacterial pneumonia involves relatively active changes caused by acute inflammatory cells in the lung compared with cancer, which replaces normal lung by growing cancer cells.

In terms of the CT setting for quantitative analysis, a previous study showed that reconstruction algorithm used affects the results of quantitative imaging analysis on CT [20]. In this study, we used 1-mm thickness images with high frequency kernel to obtain texture analysis results, and skewness and kurtosis gave different values among the ROIs even in a same lesion. This suggests that not only image thickness or reconstruction kernel but also selection bias may affect the results of texture analysis. However, the margin analysis method was quite reproducible in our study, with high ICC values.

Our study has several limitations. First, it was retrospective study at a single tertiary center. A prospectively designed larger cohort study would be necessary to confirm the preliminary results of the margin analysis method. Second, we included pneumococcal and *Klebsiella* pneumonias as they are considered common and representative of consolidative pneumonia; however, the two entities could not cover all consolidative pneumonia. Further, not only bacterial pneumonia but other diseases such as fungal infection, organizing pneumonia, and even consolidative lymphoma might mimic invasive mucinous adenocarcinoma.[3; 21] Prospective studies that focus on margin information in various pulmonary diseases are needed and we speculate that this margin analysis method could be applied to other diseases to differentiate cancer and mimickers.

## Conclusions

Using pathologically confirmed cases of invasive mucinous adenocarcinoma and bacterial pneumonia which are presenting as consolidation on CT, we investigated the transition of marginal attenuation from lesion to normal lung in the region of interest (ROI) and propose its application to discriminating between tumors and inflammatory lesions of the lung. Our results focusing on the margin of lesions could be promising as an imaging biomarker for future computer-aided diagnosis. The procedure can be performed in 1-minute using a conventional computer and open source ImageJ together with our Java software. We believe that the margin-analysis method for plotting attenuation changes will improve the differential diagnosis of invasive mucinous adenocarcinoma and bacterial pneumonia manifesting as air space consolidation. The findings of the method correspond to intuitive visual assessment of CT scans by chest radiologists. We hope to use our confirmed accumulated CT data sets on cancers and infections in a quantitative manner for machine learning and building artificial intelligence in the field of radiology.

## Supporting information

S1 File CT visual analysis findings of the patients. (XLSX)

S2 File CT margin and texture analyses results of the patients. (XLSX)
